# Maternal, Pregnancy, and Infant Outcomes in Women Treated for Multidrug-Resistant/Rifampicin-Resistant Tuberculosis With Novel and Repurposed Drugs in KwaZulu-Natal, South Africa

**DOI:** 10.1093/cid/ciaf593

**Published:** 2025-10-29

**Authors:** Marian Loveday, Jennifer Hughes, Nobuhle Mchunu, Nonhlanhla Yende-Zuma, Kerry Holloway, Sindisiwe Hlangu, Sunitha Chotoo, Nalini Singh, James A Seddon

**Affiliations:** HIV and Other Infectious Diseases Research Unit, South African Medical Research Council, Durban, South Africa; SAMRC-CAPRISA HIV-TB Pathogenesis and Treatment Research Unit, Centre for the AIDS programme of Research in South Africa, University of KwaZulu-Natal, Durban, South Africa; Desmond Tutu TB Centre, Department of Paediatrics and Child Health, Stellenbosch University, Stellenbosch, South Africa; Biostatistics Research Unit, South African Medical Research Council, Durban, South Africa; Biostatistics Research Unit, South African Medical Research Council, Durban, South Africa; MDR/RR-TB Unit, King Dinuzulu Hospital Complex, Sydenham, Durban, South Africa; HIV and Other Infectious Diseases Research Unit, South African Medical Research Council, Durban, South Africa; MDR/RR-TB Unit, King Dinuzulu Hospital Complex, Sydenham, Durban, South Africa; MDR/RR-TB Unit, King Dinuzulu Hospital Complex, Sydenham, Durban, South Africa; Desmond Tutu TB Centre, Department of Paediatrics and Child Health, Stellenbosch University, Stellenbosch, South Africa; Department of Infectious Disease, Imperial College London, London, United Kingdom

**Keywords:** tuberculosis, pregnancy, drug resistant, outcome

## Abstract

**Background:**

There is limited information on the safety and efficacy of new and repurposed second-line anti-tuberculosis (TB) drugs in pregnant women.

**Methods:**

Pregnant women initiating treatment for multidrug-resistant/rifampicin-resistant (MDR/RR) TB from 1 January 2018 to 31 March 2024 were included in this prospective, observational study. Maternal TB treatment and pregnancy outcomes were documented through ongoing record reviews and clinical assessments conducted to describe infant outcomes. Using Poisson regression with robust standard errors, we evaluated factors associated with favorable maternal treatment and pregnancy outcomes.

**Results:**

Of 70 pregnant women treated for MDR/RR TB, 53 (76%) were diagnosed with human immunodeficiency virus (HIV). Favorable TB treatment outcomes were reported in 44 (62.9%) women. Sixty-seven (95.7%) of the 70 infants were born alive;median gestational age 38 weeks (interquartile range [IQR], 37.0–39.0) and median birth weight 2970 g (IQR, 2560–3280). All infants were exposed to bedaquiline. Twenty-three (34.3%) women had unfavorable pregnancy outcomes, with 20 (29.8%) infants born prematurely and/or with low birth weight. Women with positive sputum smears at treatment initiation were less likely to have a favorable treatment outcome (adjusted risk ratio, 0.33; 95% confidence interval, .17–.66; *P* = .002). Of the 43 infants evaluated at 12 months, 27 (62.8%) had favorable outcomes, with 10 (23.3%) infants developing TB in their first year of life.

**Conclusions:**

The high TB–HIV coinfection rate is likely to have contributed to unfavorable pregnancy outcomes. Supportive maternal adherence counseling and infant TB preventive treatment are needed to prevent TB transmission to infants.

## Teaser

Despite the introduction of shorter all-oral regimens for multidrug-resistant/rifampicin-resistant tuberculosis (TB), outcomes remain suboptimal. Adherence counseling and careful management of the mother–infant pair are needed to improve maternal treatment outcomes and reduce mother-to-infant TB transmission.

Multidrug-resistant/rifampicin-resistant (MDR/RR) tuberculosis (TB) is a global public health threat, with an estimated annual incidence of 427 000 in 2023 [1]. In pregnancy and the postpartum period, women are at increased risk of TB and MDR/RR TB [2, 3], especially those with human immunodeficiency virus-1 (HIV) [4]. Maternal TB increases adverse pregnancy outcomes [5–7] and increases the risk of transmission of both *Mycobacterium tuberculosis* (*Mtb*) and HIV to their infants [8]. In turn, pregnancy increases the risks of adverse TB outcomes, including hospitalization and death [5, 6]. The last decade has seen the development of injectable-free MDR/RR TB regimens that contain novel agents. These regimens have improved treatment outcomes, are less toxic, and are much shorter, decreasing treatment duration from 24 months to 6 months. However, there is limited evidence to inform the treatment of MDR/RR TB during pregnancy with these shorter regimens, which include novel and repurposed drugs (ie, bedaquiline, delamanid, later-generation fluoroquinolones, linezolid, and clofazimine). It is therefore essential that these novel regimens be evaluated in pregnant and postpartum women.

We previously described maternal treatment, pregnancy, and infant outcomes in 108 pregnant women with MDR/RR TB treated between 1 January 2013 and 31 December 2017 [9]. In 2018, the South African MDR/RR TB treatment guidelines were revised to recommend a shorter 9- to 11-month regimen that contains bedaquiline instead of an injectable agent for patients with MDR/RR TB, including pregnant women [10]. In 2023, South Africa implemented an adapted World Health Organization (WHO) guideline, recommending an all-oral 6-month regimen for people with MDR/RR TB [11, 12]. For pregnant women, the adapted regimen contained bedaquiline, delamanid, linezolid, and levofloxacin. Here, we describe maternal treatment, pregnancy, and infant outcomes for pregnant women treated for MDR/RR TB.

## METHODS

In this prospective, observational cohort study, we enrolled pregnant women aged ≥18 years who started treatment for MDR/RR TB at King Dinuzulu Hospital (KDH). KDH is the specialist referral hospital for MDR/RR TB in KwaZulu-Natal province that pregnant women are referred to for treatment initiation and ongoing management, in line with national treatment guidelines [10, 12].

### Definitions and Study Outcomes

Definitions of baseline patient characteristics; *Mtb* drug resistance patterns; and maternal TB treatment, pregnancy, and infant outcomes are detailed in Supplementary Table 1. The evolving history of MDR/RR TB treatment recommendations for pregnant women in South Africa is tabulated in Supplementary Table 2.

We defined 3 outcomes: maternal TB treatment outcomes, pregnancy outcomes, and infant outcomes; each was considered favorable or unfavorable. Maternal TB treatment outcomes were based on WHO guidelines. Favorable outcomes were defined as either cure or treatment completion, and unfavorable treatment outcomes were defined as death, loss to follow-up (LTFU), or treatment failure [13]. LTFU was assigned to patients who had interrupted their treatment for ≥2 consecutive months [13]. Poor adherence refers to a break in treatment of <2 months.

Pregnancy outcomes were favorable if the infant was born alive, at term (≥37 weeks of pregnancy), and weighing ≥2500 g [14]. Unfavorable pregnancy outcomes comprised fetal or neonatal deaths, which included stillbirths and miscarriages, elective termination of pregnancy at any gestational age, preterm delivery (<37 weeks), or low birth weight (<2500 g).

Infants were classified as having an unfavorable outcome if, after 12 months, their growth faltered according to the *Road to Health* book (a routine record of the child's medical history, health, growth, and development), they had difficulty achieving developmental milestones, they developed TB, or they died during this period. A favorable outcome was assigned if they did not have an unfavorable outcome as defined. We used the WHO definition of growth faltering as “a fall in weight-for-age *z-*score of ≥1.0” [15]. Developmental screening was conducted in line with developmental milestones outlined in the *Road to Health* book, and developmental delay was defined as a significant delay in 2 or more of the following developmental domains: gross/fine motor, speech/language, social/personal, cognition, and activities of daily living [16]. All infants with signs and symptoms of TB and started on TB treatment during the follow-up period were regarded as having incident TB disease.

### Data Variables and Collection

Maternal medical records were reviewed to collect demographic, clinical, and laboratory data at treatment initiation and to capture women's responses to treatment. At the end of treatment, maternal MDR/RR TB treatment outcomes were assigned by the researchers according to standard WHO definitions. Medical records were reviewed until women had attended their routine 6-month post-treatment follow-up appointment. Pregnancy outcomes and birth weight were extracted from patient-held notes and the *Road to Health* books. Infants were assessed clinically by the study pediatrician at KDH at ages 6 weeks, 6 months, and 12 months to determine if they were thriving and developing normally and screened for symptoms and signs of TB disease. An infant outcome was assigned at the 12-month assessment. If any problems were detected, the child was referred to routine care for further evaluation and investigation.

### Data Management and Statistical Analyses

Categorical clinical characteristics, including maternal TB treatment, pregnancy, and infant outcomes, were summarized using frequencies and percentages. Quantitative characteristics were summarized using means with standard deviations or medians with interquartile ranges (IQRs) where appropriate. Univariable and multivariable Poisson regression models with robust standard errors were used to identify factors associated with favorable maternal TB treatment and pregnancy outcomes. The models were adjusted for potential confounders, including age, MDR/RR TB resistance pattern, sputum smear microscopy, hemoglobin level, baseline viral load, and trimester when MDR/RR TB treatment was initiated. The trimester of treatment initiaion was treated as the main exposure variable because of it potential influence on drug exposure. Given the small sample size, model overfitting and residual confounding were avoided by selecting clinically important variables in the multivariable model, regardless of their statistical significance in the univariable model. This approach was particularly important given our small sample size, where the inclusion of all the covariates in the multivariable model led to model instability/overfitting; hence, the inclusion of a few clinically relevant predictors was necessary. Results were presented as adjusted risk ratios (aRRs) with corresponding 95% confidence intervals (CIs). All analyses were conducted using Stata software version 18.0 (StataCorp, 2023. Stata Statistical Software: Release 18. College Station, Texas).

### Ethics

The South African Medical Research Council Ethics Review Committee and the KwaZulu-Natal Health Research Committee approved the study.

## RESULTS

### Baseline Characteristics

**Figure 1.**
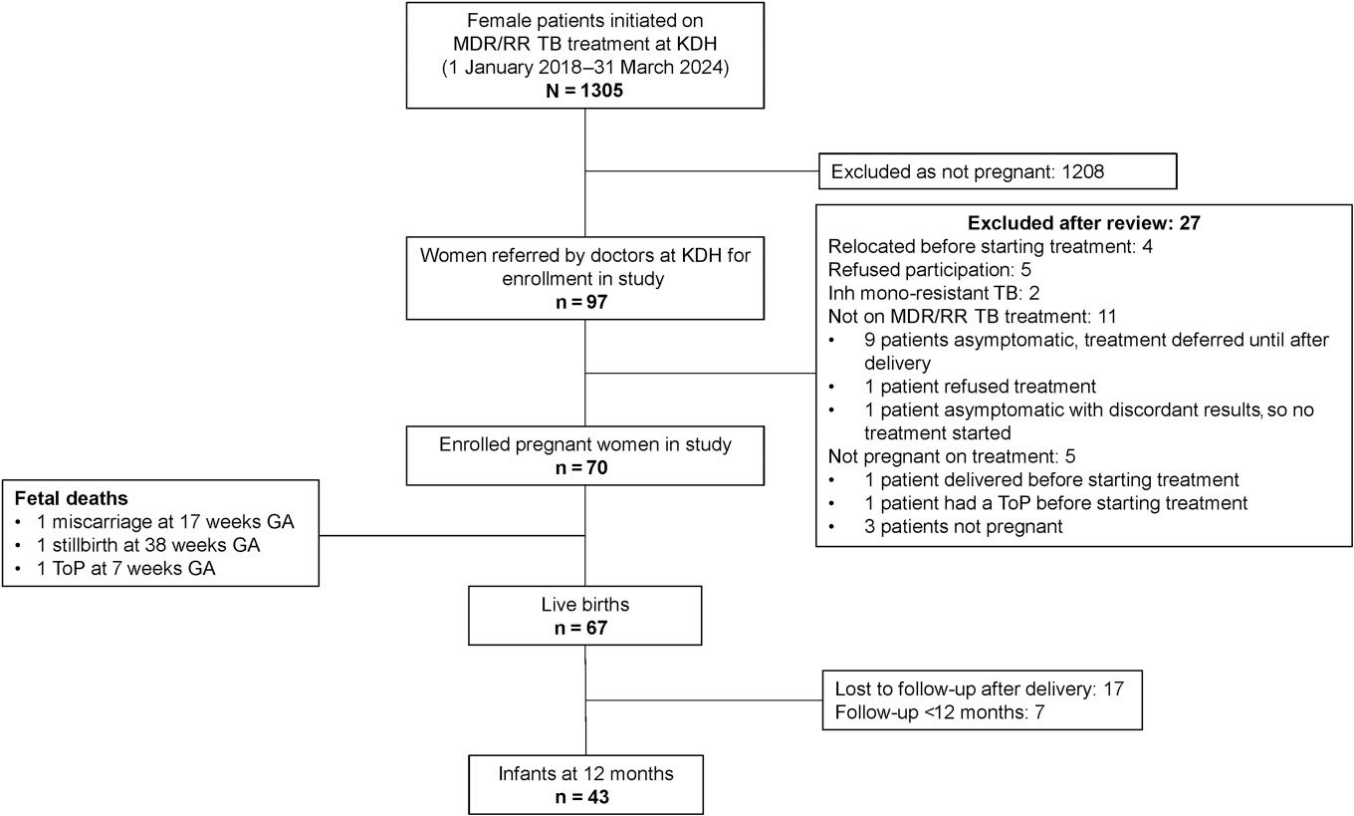
Schematic of enrollment and attrition (1 January 2018–31 March 2024). Abbreviations: GA, gestational age; KDH, King Dinuzulu Hospital; MDR/RR, multidrug-resistant/rifampicin-resistant; TB, tuberculosis; ToP, termination of pregnancy; Inh, isoniazid.

Between 1 January 2018 and 31 March 2024, 1305 females of reproductive age (15–45 years) were initiated on treatment for MDR/RR TB or pre-extensively drug-resistant (pre-XDR) TB/extensively drug-resistant (XDR) TB (see Supplementary Table 1 for definitions), of whom 97 were reported to be pregnant and referred for study screening. Twenty-seven women were excluded (Figure 1). Seventy women who received MDR/RR TB treatment for ≥14 days while pregnant were included in this analysis, of whom 53 (75.7%) were diagnosed with HIV (Table 1). Sixty-two (88.6%) women were known to be pregnant prior to starting MDR/RR TB treatment, and the median gestational age at the start of treatment was 21.9 weeks (IQR, 15.9–26.1). At treatment initiation, sputum culture results were available for 64 women, of whom 51 (78.5%) had positive sputum cultures. Of the 6 women for whom culture results were not available, 2 were contaminated and 4 records were not recorded, either because the culture was not done or the result was not recorded. Forty-six (65.7%) women received a short (≤11 months) all-oral regimen, none of whom received an injectable. Of those who received a short regimen, 11 (16%) received a 6-month regimen with bedaquiline, delamanid, linezolid, and levofloxacin/clofazimine (Supplementary Table 2).

**Table 1.** Clinical Characteristics of Pregnant Women at Multidrug-Resistant/Rifampicin-Resistant Tuberculosis Treatment Initiation (N = 70)

Clinical Characteristics	No. (%)
Age, years: mean; SD	29.1; 6.8
Hemoglobin, g/dL: mean; SD (n = 67)	10.0; 1.9
BMI, kg/m^2^: mean; SD (n = 68)	24.7; 5.8
BMI categories, kg/m^2^ (n = 68)	
Underweight (<18.5)	7 (10.3%)
Normal (18.5–24.99)	29 (42.6%)
Overweight (25–29.99)	24(35.3%)
Obese (≥30)	8 (11.8%)

TB characteristics	
Sputum smear-positive at treatment initiation (n = 68)	33 (48.5%)
Culture-positive at treatment initiation (n = 64)	51 (78.5%)
Previous TB or MDR/RR TB (n = 67)	31 (46.3%)
Site of TB: Pulmonary (n = 65)	61 (93.8%)
Chest radiograph (n = 64)	
Extensive disease^a^	32 (50.0%)
Minimal	24 (37.5%)
Normal	8 (12.5%)
Resistance pattern^b^	
RR/Rif-mono/MDR TB	56 (80.0%)
Pre-XDR/XDR TB	14 (20.0%)
Bedaquiline-resistance (n = 8)	2 (25.0%)

HIV characteristics	
Diagnosed with HIV	53 (75.7%)
HIV-positive patients on ART before MDR/RR TB treatment started (n = 53)	47 (90.4%)
Started ART within 1 month of MDR/RR TB treatment initiation	3 (5.8%)
Started ART >1 month after MDR/RR TB treatment initiation	2 (3.8%)
CD4 count, cells/mm^3^ median; IQR (n = 52)	402.5; 220.0–660.5
Viral load (n = 46)	
Undetectable (<40 copies/mL)	29 (63.0%)

Pregnancy characteristics	
Pregnant before MDR/RR TB treatment started	62 (88.6%)
Gestational age at treatment start, weeks: median; IQR (n = 61)	21.9; 15.9–26.1

Abbreviations: ART, antiretroviral therapy; BMI, body mass index; HIV, human immunodeficiency virus; IQR, interquartile range; MDR/RR, multidrug-resistant/rifampicin-resistant; pre-XDR, preextensively drug-resistant; rif-mono, rifampicin-monoresistant; SD, standard deviation; TB, tuberculosis; XDR, extensively drug-resistant.

^a^Extensive disease was classified as bilateral disease and/or cavities on chest radiograph.

^b^Definitions of drug resistance in line with World Health Organization 2020 definitions and outlined in Supplementary Table 1.

### Drug Exposure

The median maternal treatment duration was 280 days (IQR, 174.0–408.0), and the median fetal drug exposure was 103 days (IQR, 69.0–153.5; Table 2). Sixteen (22.9%) women were initiated on treatment during or before the first trimester. All received bedaquiline-based regimens, 89.7% received linezolid, 82.9% received later-generation fluoroquinolones and clofazimine, 42 (60%) received pyrazinamide, and 37 (52.9%) received ethambutol. Only 24 (34.3%) women received a regimen that contained delamanid.

**Table 2.** Maternal Multidrug-Resistant/Rifampicin-Resistant Tuberculosis Treatment Received and Infant Exposure

Maternal Treatment Received and Infant Drug Exposure	Maternal (N = 70)	Infant (N = 69)^a^
Maternal treatment duration, days: median; IQR	280.0; 174.0–408.0	…
Fetal drug exposure duration, days: median; IQR	…	103.00; 69.0–153.5
Trimester when MDR/RR TB treatment was initiated	…	…
During or before first trimester	16 (22.9%)	…
Initiated in second trimester	37 (52.9%)	…
Initiated in third trimester	17 (24.3%)	…
Fluoroquinolones (later generation), no. (%)	58 (82.9)	57 (82.6)
Fluoroquinolone treatment duration, days: median; IQR	220; 168.0–316.0	97.0; 66.0–136.0
Bedaquiline, no. (%)	70 (100.0)	69 (100.0)
Bedaquiline treatment duration, days: median; IQR	193.0; 161.0–273.0	97.0; 63.0–120.0
Clofazimine, no. (%)	58 (82.9)	57 (82.6)
Clofazimine treatment duration, days: median; IQR	301.0; 210.0–523.0	97.0; 62.0–140.0
Linezolid, no. (%)	61 (89.7)	60 (87.0)
Linezolid treatment duration, days: median; IQR	140.0; 62.0–196.0	71.5; 46.5–109.0
Delamanid, no. (%)	24 (34.3)	21 (30.4)
Delamanid treatment duration, days: median [IQR]	181.5; 168.5–215.5	93.0; 66.0–136.0

Abbreviations: IQR, interquartile range; MDR/RR, multidrug-resistant/rifampicin-resistant; TB, tuberculosis.

^a^One infant was excluded from the exposure analysis due to an elective termination of pregnancy at 7 weeks’ gestational age.

### Maternal TB Treatment Outcomes

Forty-four (62.9%) women had favorable treatment outcomes (Table 3). Women whose sputum was smear-positive at the start of treatment were less likely to have a favorable treatment outcome (aRR, 0.33; 95% CI, .17–.66; *P* = .002) compared with women whose smears were negative (Table 4). Of the 46 women who received a short all-oral regimen, 23 (50.0%) had a favorable treatment outcome, 18 (39.1%) were LTFU, 4 failed treatment, and 1 died.

**Table 3.** Maternal Tuberculosis Treatment, Pregnancy, and Infant Outcomes

Primary Outcomes	Outcome Variables	1st Trimester Drug Exposure (n = 16)	Second Trimester Drug Exposure (n = 37)	Third Trimester Drug Exposure (n = 17)	Total (N = 70)
Maternal treatment outcomes	**Favorable treatment outcomes**	**8** (**50.0%)**	**25** (**67.6%)**	**11** (**64.7%)**	**44** (**62.9%)**
	Cure	7 (43.8%)	18 (48.6%)	8 (47.1%)	33 (47.1%)
	Complete	1 (6.3)	7 (18.9%)	3 (17.6%)	11 (15.7%)
	**Unfavorable treatment outcomes**	**8** (**50.0%)**	**12** (**32.4%)**	**6** (**35.3%)**	**26** (**37.1)**
	Lost to follow-up	6 (37.5%)	9 (24.3%)	5 (29.4%)	20 (28.6%)
	Death	1 (6.3%)	0	0	1 (1.4%)
	Treatment failed	1 (6.3%)	3 (8.1%)	1 (5.9%)	5 (7.1%)
Pregnancy outcomes	Number of deliveries	16	37	17	70
	Live births	14 (8. 5%)	36 (97.3%)	17 (100.0%)	67 (95.7%)
	Gestational age at delivery, wk: median; IQR	37.5 (33.5–38.5)	38.0; 37.0–39.0	39.0; 38.0–40.0	38.0; 37.0–39.0
	Birth weight, g: median; IQR	3000; 2600–3240	3000; 2275–3375	2810; 2700–3120	2970; 2560–3280
	Fetal deaths	2 (12.5%)	1 (2.7%)	0	3 (4.3%)
	Stillbirth	0	1 (2.7%)	0	1 (1.4%)
	Miscarriage	1 (6.3%)	0	0	1 (1.4%)
	Termination of pregnancy	1 (6.3%)	0	0	1 (1.4%)
	**Favorable pregnancy outcomes^a^**	**9 (56.2%)**	**22 (58.3%)**	**16 (94.1%)**	**47 (67.1%)**
	**Unfavorable pregnancy outcomes^b^**	**7 (43.8%)**	**15 (40. 5%)**	**1 (5.6%)**	**23 (32.9%)**
	Fetal deaths	2 (12.5%)	1 (2.7%)	0	3 (4.5%)
	Premature/low birth weight	5 (31.3%)	14 (37.8%)	1 (5.6%)	20 (29.8%)
	<37 weeks	5 (35.7%)	8 (21.6%)	1 (5.6%)	14 (20.9%)
	Birth weight <2500 g	3 (21.4%)	11 (29.7%)	0	14 (20.9%)
Infant outcomes	No infant outcomes at 12 months	**7** (**43.8%)**	**13** (**35%)**	**7** (**47.4%)**	**27** (**41.8%)**
	Fetal deaths, so no infant outcome	2 (12.5%)	1 (2.8%)	0	3 (4.8%)
	Lost to follow-up after birth	3 (18.8%)	8 (22.2%)	6 (33.3%)	17 (25.4%)
	Follow-up <12 months	2 (12.5%)	4 (10.8%)	1(11.1%)	7 (11.9%)
	*Infant outcomes (favorable and unfavorable)*	*9*	*24*	*10*	*43 (61.4%)*
	**Favorable infant outcomes at 12 months^c^**	**6** (**66.7%)**	**16** (**66.7%)**	**5** (**50%)**	**27** (**62.8%)**
	Thriving normally	8 (88.9%)	16 (66.7%)	7 (70%)	31 (72.1%)
	Normal development	8 (88.9%)	22 (91.75%)	10 (100%)	39 (93.0%)
	**Unfavorable infant outcomes at 12 months ^d^**	**3** (**33.3%)**	**8** (**18.6%)**	**5** (**50%)**	**16** (**37.2%)**
	Failure to thrive	1 (11.1%)	7 (29.2%)	3 (30%)	11 (25.6%)
	Delayed development	1 (11.1%)	1 (4.2.0%)	0	2 (2.8%)
	Neonatal death	0	1 (4.2%)	0	1 (1.4%)
	Developed tuberculosis in the first year of life	2 (22.2%)	4 (16.6%)	4 (40%)	10 (23.3%)
	Laboratory diagnosis	1 (11.1%)	2 (8.3%)	1 (10%)	4 (9.3%)
	Clinical diagnosis	1 (11.1%)	2 (8.3%)	3 (30%)	6 (14.0%)

Abbreviations: g, grams; IQR, interquartile range.

Favourable and unfavourable outcomes are marked in bold as these are the primary outcomes of the study.

^a^A favorable pregnancy outcome is defined as an infant born at term (≥37 weeks) with a birth weight ≥2500 g.

^b^An unfavorable pregnancy outcome is defined as an infant born preterm (<37 weeks) or with a low birth weight (<2500 g).

^c^A favorable infant outcome is defined as an infant who, at 12 months, is thriving, that is, gaining weight and following the normal trajectory according to the growth chart.

^d^An unfavorable infant outcome is defined as an infant who, at 12 months, failed to maintain an established pattern of growth, or who reached their developmental milestones later than the average infant, or who was treated for tuberculosis (TB) following a laboratory diagnosis of TB or multidrug-resistant/rifampicin-resistant TB, or who had the signs and symptoms of active TB disease.

**Table 4.** Predictors of Favorable Maternal Tuberculosis Treatment Outcomes

Clinical Characteristics at Treatment Start	Unadjusted Risk Ratio (95% CI)	*P* Value	Adjusted Risk Ratio (95% CI)	*P* Value
Age	0.97 (.84–1.12)	.711	0.92 (.78–1.07)	.275
Previous TB: Ref (no TB)				
Previous TB	0.99 (.67–1.45)	.949	0.88 (.52–1.49)	.633
Resistance pattern: Ref (pre-XDR/XDR TB)				
MDR/RR TB/Rif-mono TB	1.28 (.73–2.26)	.388	1.15 (.65–2.07)	.636
More extensive disease on chest X ray: Ref (clear/minimal changes)				
Extensive disease^a^	1.03 (.68–1.58)	.879	1.11 (.66–1.87)	.702
Sputum smear at diagnosis: Ref (negative)				
Smear positive	0.51 (.32–.81)	.004	0.33 (.17–.66)	.002
Culture at treatment initiation: Ref (negative)				
Culture positive	0.77 (.51–1.17)	.226		
Hemoglobin at diagnosis, g/dL: Ref (normal ≥8)				
Abnormal (<8)	1.10 (.60–2.03)	.748	1.08 (.60–1.97)	.792
Body mass index, kg/m^2^: Ref (normal, 18.5–24.99)				
Underweight (<18.5)	0.80 (.32–2.03)	.638	…	
Overweight (25–29.99/Obese≥30)	1.31 (.86–1.99)	.212	…	
HIV status: Ref (HIV-negative)				
HIV-positive	1.40 (.81–2.42)	.225	…	
CD4 count, cells/mm^3^: Ref (HIV-negative)				
<200	0.89 (.38–2.06)	.778	…	
≥200	1.61 (.94–2.76)	.085	…	
Baseline viral load: Ref (HIV-negative)				
Undetectable (<40 copies/mL)	1.63 (.94–2.84)	.080	1.73 (.91–3.32)	.097
Detectable (≥ 40 copies/mL)	1.13 (.57–2.22)	.734	1.40 (.72–2.73)	.320
ART before RR TB treatment: Ref (HIV-negative)				
On ART prior to MDR/RR TB treatment start	1.45 (.84–2.50)	.184		
Started ART after MDR/RR TB treatment started	1.28 (.53–3.08)	.589	…	
Trimester when MDR/RR TB treatment was initiated: Ref (third trimester)				
First trimester	0.77 (.42–1.42)	.405	1.08 (.61–1.88)	.800
Second trimester	1.00 (.65–1.54)	1.000	0.74 (.49–1.13)	.169

Abbreviations: ART, antiretroviral therapy; CI, confidence interval; HIV, human immunodeficiency virus; MDR/RR, multidrug-resistant/rifampicin-resistant; pre-XDR, preextensively drug-resistant; rif-mono, rifampicin-monoresistant; Ref, reference; RR, rifampicin-resistant; TB, tuberculosis; XDR, extensively drug-resistant.

^a^Extensive disease was classified as bilateral disease and/or cavities on chest radiograph.

Of all women, including those who received a long regimen, 20 (28.6%) were LTFU, 4 died following disengagement from care and the delivery of their infants. One woman died 60 days after her LTFU classification. The remaining 3 all restarted treatment after a LTFU classification but continued to have periods of suboptimal adherence before dying. One woman died a year after her initial LTFU classification, the second and third died two years and 3.5 years after their LTFU classifications respectively.

In addition to the women LTFU, poor adherence was reported in the 5 women in whom treatment failed and in the woman who died. The woman who died was diagnosed with HIV and had fluoroquinolone-resistant MDR/RR TB. She died after 310 days of MDR/RR TB treatment, 26 days after the delivery of her preterm infant who was born at 35 weeks’ gestational age and weighed 1920 g. Of the 5 women for whom treatment failed, the median number of days to treatment failure was 301 (IQR, 161–377). Two of these women had XDR TB.

### Pregnancy Outcomes

**Figure 2.**
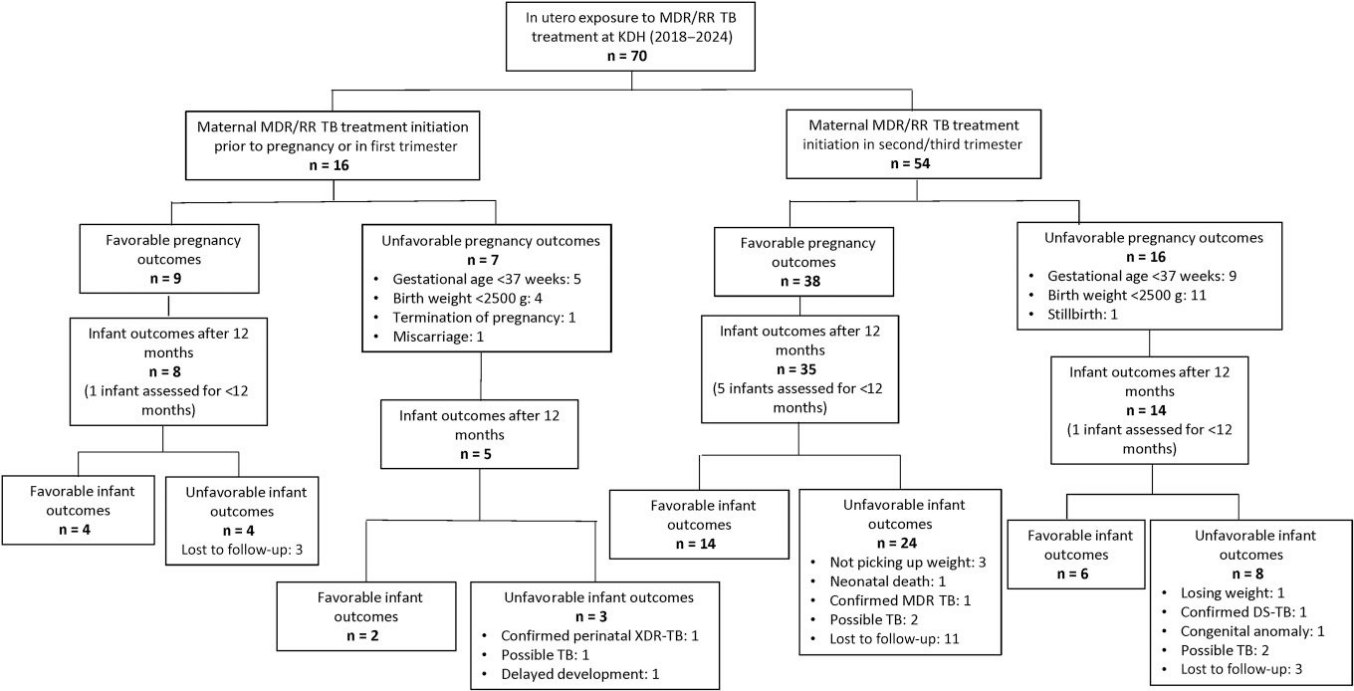
Pregnancy and infant outcomes by trimester of exposure (first vs second and third trimesters). Abbreviations: DS, drug-susceptible; KDH, King Dinuzulu Hospital; MDR/RR, multidrug-resistant/rifampicin-resistant; TB, tuberculosis; XDR, extensively drug-resistant.

Of the 70 pregnancies, 67 infants (95.7%) were born alive, with a median gestational age of 38.0 weeks (IQR, 37.0–39.0) and median birth weight of 2970 g (IQR, 2560–3280; Table 3). Forty-seven (67.1%) of the pregnancies had a favorable outcome. There were 3 fetal and neonatal deaths, and 20 (29.8%) of the infants were born before 37 weeks’ gestational age and/or with a low birth weight. Of the 23 unfavorable pregnancy outcomes, 20 (87%) of the women were diagnosed with HIV. There was no association between disease severity at baseline (ie, women who at treatment initiation had smear-positive sputum, extensive TB disease on chest X ray, or history of prior TB) and a higher risk of an unfavorable pregnancy outcome (Table 5). However, women who started MDR/RR TB treatment in the first or second trimester had a 39% (aRR, 0.61; 95% CI, .38–.98; *P* = .039) and 35% (aRR, 0.65; 95% CI, .43–.97; *P* = .035) lower likelihood of a favorable pregnancy outcome, respectively, compared with women who started treatment in the third trimester (Table 5, Figure 2).

**Table 5.** Predictors of Favorable Pregnancy Outcomes

Clinical Characteristics at Treatment Start	Unadjusted Risk Ratio (95% CI)	*P* Value	Adjusted Risk Ratio (95% CI)	*P* Value
Age	0.97 (.86–1.10)	.677	1.00 (.86–1.17)	.988
Previous TB: Ref (no TB)				
Yes	0.62 (.42–.92)	.018	0.67 (.41–1.08)	1.00
Resistance pattern: Ref (pre-XDR/XDR TB)				
RR/MDR/Rif-mono TB	1.05 (.68–1.62)	.838	1.01 (.65–1.56)	.975
More extensive disease on chest X ray: Ref (clear/minimal changes)				
Extensive disease^a^	0.70 (.48–1.02)	.062	0.94 (.52–1.71)	.855
Sputum smear at diagnosis: Ref (negative)				
Smear-positive	0.63 (.43–.90)	.013	0.80 (.51–1.26)	.341
Culture at treatment initiation: Ref (negative)				
Culture-positive	0.81 (.58–1.15)	.246		
Hemoglobin at diagnosis, g/dL: Ref (normal ≥8)				
Abnormal (<8)	0.84 (.43–1.65)	.617	0.64 (.31–1.31)	.220
Body mass index, kg/m^2^: Ref (normal 18.5–24.99)				
Underweight (<18.5)	0.49 (.14–1.65)	.248	…	
Overweight (25–29.99/Obese≥30)	1.43 (1.01–2.02)	.042	…	
HIV status: Ref (HIV-negative)				
HIV-positive	0.75 (.55–1.02)	.065	…	
CD4 count, cells/mm^3^: Ref (HIV negative)				
<200	0.61 (.33–1.12)	.110	…	
≥200	0.78 (.56–1.08)	.130	…	
Baseline viral load: Ref (HIV-negative)				
Undetectable (<40 copies/mL)	0.84 (.60–1.17)	.294	1.12 (.61–2.05)	.725
Detectable (≥40 copies/mL)	0.68 (.42–1.11)	.126	1.09 (.67–1.76)	.725
ART before RR TB treatment: Ref (HIV-negative)				
On ART prior to RR TB treatment start	0.77 (.56–1.05)	.096	…	
Started ART after RR TB treatment started	0.49 (.16–1.46)	.200	…	
Trimester when MDR/RR TB treatment was initiated: Ref (third trimester)				
First trimester	0.57 (.35–.93)	.024	0.61 (.38–.98)	.039
Second trimester	0.63 (.47–.85)	.002	0.65 (.43− .97)	.035

Abbreviations: ART, antiretroviral therapy; CI, confidence interval; HIV, human immunodeficiency virus; MDR/RR, multidrug/rifampicin-resistant; pre-XDR, preextensively drug-resistant; rif-mono, rifampicin-monoresistant; Ref, reference; RR, rifampicin-resistant; TB, tuberculosis; XDR, extensively drug resistant.

^a^Extensive disease was classified as bilateral disease and/or cavities on chest radiograph.

### Infant Outcomes

We followed 43 of 67 live infants at 12 months, but were unable to follow up on 24 infants; 7 had not reached the 12-month follow-up assessment, and 17 could not be found or contacted, despite multiple attempts to contact the family (Table 3). Favorable infant outcomes were reported in 27 (61.4%) of the 43 live infants who were followed. There was an early neonatal death at 27 days due to a congenital cardiac condition. Another infant was diagnosed with genu recurvatum at birth.

Ten (23.3%) of the 43 infants were initiated on TB treatment in their first year of life. The mothers of 7 of these infants had positive cultures at infant diagnosis; 4 reported suboptimal adherence over the intrapartum period, 1 had been on treatment for only a month, and 2 were inappropriately treated at their local primary healthcare clinic despite laboratory-confirmed MDR/RR TB. Six infants had the signs and symptoms of TB but no microbiological confirmation. Four had microbiologically confirmed TB, 3 with confirmed MDR/RR TB and 1 with confirmed drug-susceptible TB. Given the suboptimal maternal adherence and treatment, culture positivity, drug susceptiblity testing (DST) concordance, and age of the infants (all aged ≤3 months and in close contact with their mother), we hypothesized that perinatal transmission was responsible for their TB. Nine infants started MDR/RR TB treatment. One 12-month-old infant whose mother had successfully completed MDR/RR TB treatment 6 months previously started first-line TB treatment following household exposure to another adult with drug-susceptible TB.

## DISCUSSION

Previously, we reported favorable maternal TB treatment outcomes in 67% of 108 pregnant women treated for MDR/RR TB and favorable pregnancy and infant outcomes in 52% and 84% of the cohort, respectively [9]. In the current cohort of 70 women, 62.9% had favorable treatment outcomes; favorable pregnancy outcomes were reported in 66.1%, and 63.4% of the infants assessed at 12 months had a favorable infant outcome. In our 2013–2018 cohort, only 52% had a favorable pregnancy outcome, as more than a third of the infants (35%) had low birth weight, with low birth weight reported in more infants exposed to bedaquiline compared with those not exposed (45% vs 26%; *P* = .034) [9]. The increase in favorable pregnancy outcomes in the current cohort is encouraging (low birth weight was reported in only 20.9% of infants), suggesting that the association we reported previously between bedaquiline exposure and low birth weight is not supported. However, this may partly reflect temporal changes in standard of care.

In our cohort of 70 women treated with novel and repurposed drugs, 44 (62.9%) had favorable treatment outcomes. In the TB-PRACTECAL trial, 16 women treated with novel regimens that contained bedaquiline, pretomanid, and linezolid became pregnant during the study; all had a successful treatment outcome [17]. In the endTB and STEM-TB studies, 43 pregnant women were treated with new or repurposed drugs, and 42 (98%) had a successful treatment outcome [18]. Fewer participants in our study (44, 62.9%) had favorable treatment outcomes compared with those reported in these trials. This could be due to the high proportion of women (53, 75.7%) diagnosed with HIV, together with the operational setting of our study, where 20 (28.6%) women were LTFU.

Over the last decade, clinical trials of shorter all-oral, less toxic MDR/RR TB regimens have been shown to improve treatment outcomes considerably, with favorable treatment outcomes of >75% reported in the Bangladesh, Stream, Nix, and ZeNix studies [19–22]. Given the performance of these shorter regimens in clinical trials, similar increases in favorable treatment outcomes were anticipated in operational settings; however, this has not been the case in South Africa. In 2018, after the introduction of a 9- to 11-month all-oral regimen, favorable treatment outcomes were reported in 64% of the national cohort, mortality in 20%, and LTFU for 15%, with similar findings reported from a rural setting [23, 24]. A recent study reported 38% favorable outcomes in patients treated with the 9- to 11-month all-oral regimen, with nearly one third of patients having periods of treatment interruption of ≥2 consecutive months and 35% switching to an individualized regimen [25]. The lower proportion of favorable treatment outcomes we report in the current cohort compared with the previous cohort is due, in part, to the higher proportion of women LTFU, 28.6% vs 23%. This increase in LTFU is disappointing, as two thirds of the women LTFU received a 9- to 11-month regimen. This limited evidence suggests that all-oral regimens in pregnant women have not led to the anticipated reductions in LTFU and improvements in treatment outcomes. It may be that with the introduction of these all-oral regimens, hospital admission time has decreased and medical teams have less time to counsel and support patients about the importance of adherence.

Sixty-seven (95.7%) women in our cohort delivered a live infant, and 47 (67.1%) of pregnancy outcomes were favorable. The pregnancy outcomes we report compare favorably with those of the TB-PRACTECAL, endTB observational, and STEM-TB studies. In TB-PRACTECAL, of 16 pregnant participants, 14 pregnancy outcomes were reported. Besides 4 terminations, there were 10 live births, with normal birth weight reported for the 8 neonates for whom it was documented [17]. In the 43 pregnant women included in the endTB and STEM-TB studies, birth weight and gestational age were known for 22 women; 59% of the infants were born at term, 68% with normal birth weight [18]. Several studies have documented increased unfavorable pregnancy outcomes in women with advanced pulmonary disease or more serious manifestations of extrapulmonary disease [26–28]. Our data did not show a relationship between disease severity at baseline and a higher risk of an unfavorable pregnancy, but our study may have been underpowered to detect such differences. We did demonstrate that treatment initiation in the first or second trimester was associated with a worse pregnancy outcome compared with starting treatment in the third trimester.

Favorable infant outcomes were reported in 62.8% of the infants we followed for 12 months, which is less than the 84% we reported previously [9]. However, a larger proportion of infants in this cohort were not assessed. The proportion of infants initiated on TB treatment in the first year of life is also greater than we reported previously [9]. Recent improvements in infant screening, detection of TB disease, and appropriate initiation of TB treatment may have contributed to the increased number of infants treated for TB in the current cohort compared with our previous cohort.

It is critical to determine the factors associated with adherence and retention in care during the peripartum period for the development of appropriate interventions to ensure the health and well-being of mother–infant pairs, particularly in our setting with high rates of MDR TB and HIV coinfection. The importance of comprehensive patient-centered care and support for pregnant and postpartum women has been highlighted in a recent analysis of the Brazilian TB National Surveillance data, which showed a significant association between pregnancy and poor treatment outcomes [29].

Our relatively small study population remains a limitation for identifying predictors of unfavorable outcomes. The study was performed at a single center, limiting its generalizability. Furthermore, as it was an observational study conducted in the public sector with limited resources, the data routinely documented by health workers (eg, adherence data) were at times incomplete and of poor quality. Incomplete adherence data constrained our ability to examine its role in evaluating TB treatment outcomes. This is an important gap that future studies with larger cohorts should address. Although the proportion of infants who attended the 12-month follow-up was lower than expected, this proportion is better than in most child health programs in sub-Saharan Africa [30, 31]. The number of infants diagnosed with TB might represent under- or overdiagnosis, given the limitations of infant TB diagnostics and the high proportion of infants LTFU.

Our data suggest that new and repurposed drugs and shorter MDR/RR TB regimens have not improved treatment outcomes. Given the increased risk of TB transmission to infants when maternal adherence is suboptimal and the severe consequences thereof, it is critical to consider how perinatal maternal TB care can be enhanced, adherence optimized, and LTFU reduced so that infants, together with the rest of the family, are protected against the transmission of MDR/RR TB.

## Supplementary Material

ciaf593_Supplementary_Data

## Supplementary Data

Supplementary materials are available at *Clinical Infectious Diseases* online. Consisting of data provided by the authors to benefit the reader, the posted materials are not copyedited and are the sole responsibility of the authors, so questions or comments should be addressed to the corresponding author.
