# Supplementary material for: Maternal, Pregnancy, and Infant Outcomes in Women Treated for Multidrug-Resistant/Rifampicin-Resistant Tuberculosis With Novel and Repurposed Drugs in KwaZulu-Natal, South Africa
Source: Clin Infect Dis. 2025 Oct 29;82(5):e1053–62. doi: 10.1093/cid/ciaf593 (PMC13189659; doi:10.1093/cid/ciaf593)
Supplement: ciaf593_Supplementary_Data [file ciaf593_supplementary_data.docx]

**SUPPLEMENTARY MATERIAL**

**Supplementary Table 1: Definition of terms**

| **Clinical characteristics at treatment initiation** | |
| --- | --- |
| Pulmonary tuberculosis | Pulmonary tuberculosis was defined as disease affecting the lungs only. |
| Extensive disease | Extensive disease was defined as the presence of either cavities or disease in both left and right lung fields. |
| Patients on ART | Patients on ART before MDR/RR-TB treatment started were defined as those receiving ART for at least 30 days prior to MDR/RR-TB treatment initiation. |
| Baseline CD4 count | Baseline CD4 count was defined as the date recorded in the clinical notes closest to the date of MDR/RR-TB treatment initiation. |
| Gestational age | Gestational age was recorded from clinical notes; determined by ultrasound, dates, or both. |
| **Drug resistance definitions^1,2^** | |
| Multidrug/rifampicin resistant TB (MDR/RR-TB) | MDR/RR-TB was classified as TB caused by *Mycobacterium tuberculosis (Mtb)* with genotypic or phenotypic resistance to rifampicin. It included MDR-TB (resistance to both isoniazid and rifampicin), rifampicin mono-resistant tuberculosis (susceptibility to isoniazid), and forms of disease where rifampicin resistance has been identified, but no result for isoniazid is available. |
| Pre-extensively drug-resistant TB (Pre-XDR-TB) | Pre-XDR-TB was classified as TB disease caused by strain of *Mtb* that met the definition of MDR/RR-TB with additional resistance to any fluroquinolone. |
| Extensively drug-resistant TB (XDR-TB) | XDR-TB was classified as TB disease caused by strain of *Mtb* that met the definition of MDR/RR-TB with additional resistance to at least one fluoroquinolone (levofloxacin or moxifloxacin) and to at least one additional Group A drug either bedaquiline of linezolid. |
| New and repurposed drugs | New and repurposed drugs includes: clofazimine, bedaquiline, linezolid, delamanid and later generation fluoroquinolones (levofloxacin, moxifloxacin) |
| **Maternal TB Treatment outcomes^2,3^** | |
| **Favourable treatment outcomes** | |
| Cured | A patient with pulmonary TB with bacteriologically confirmed TB at the beginning of treatment who completed treatment as recommended by the national policy, with evidence of bacteriological response and no evidence of failure.   - “Bacteriological response” refers to bacteriological conversion with no reversion. - “Bacteriological conversion” describes a situation in a patient with bacteriologically confirmed TB where at least two consecutive cultures taken at least 7 days apart, are negative. - “Bacteriological reversion” describes a situation where at least two consecutive cultures taken at least 7 days apart, are positive either after the bacteriological conversion or in patients without bacteriological confirmation of TB. - The patient should have at least 3 negative TB cultures during the entire duration of treatment. |
| Treatment completed | A patient who has TB culture converted, has two negative TB cultures during treatment, who completed treatment as recommended by the national policy with no evidence of clinical deterioration. |
| Treatment success | The sum of cured and treatment completed. |
| **Unfavourable treatment outcomes** | |
| Treatment failure | Treatment regimen terminated or permanently regimen changed to a new regimen or treatment strategy (change of 2 or more drugs) for any of the following reasons: 1) no clinical response and/or no bacteriological response; 2) adverse drug reactions; or 3) evidence of additional drug resistance to medicines in the regimen. |
| Died | A patient who dies for any reason during treatment. |
| Lost to follow-up | A patient whose treatment was interrupted for 2 consecutive months or more. |
| Not evaluated | A patient recorded in the treatment register who does not have the necessary recorded data to enable classification of any outcome. |
| Treatment unsuccessful | The sum of treatment failure, died, lost to follow-up and not evaluated. |
| **Pregnancy outcomes** | |
| **Favourable pregnancy outcomes** – all of the features below are required to classify a pregnancy outcome as favourable | |
| Full term | Babies born ≥37 weeks of pregnancy. |
| Normal birthweight | Birth weight of ≥2500 grams according to the World Health Organization (WHO).^4^ |
| Alive | A baby born alive which lives for >28 days. |
| **Unfavourable pregnancy outcomes** – any of the following classify a pregnancy as having an unfavourable outcome | |
| Preterm birth | Babies born <37 weeks of pregnancy. |
| Miscarriage | Spontaneous loss of a pregnancy before the foetus has reached viability at 24 weeks. This includes all pregnancy losses from the time of conception until 23 completed weeks of gestation.^5^ |
| Stillbirth | In South Africa, the legal definition of stillbirth is an infant born dead after ‘6 months of intra-uterine life’ (i.e. 28 weeks since the start of the last period or 26 weeks since conception). If the gestational age is not known, a weight of 1000 g is used to legally define a stillbirth.  Infants that are born dead before this time are legally regarded as miscarriages |
| Termination of pregnancy | Termination of pregnancy is when a woman decides to end her pregnancy before the full term by medical means.  The woman must be under 13 weeks pregnant to end the pregnancy without giving reasons. If she is between 13 and 20 weeks pregnant, the pregnancy may be terminated only under specific conditions. If she is more than 20 weeks pregnant, it will be done only if her or the foetus' life is in danger or there are likely to be serious birth defects.^6^ |
| Low birthweight | A birthweight of less than 2500g (up to and including 2499g), as per the World Health Organization (WHO).^4^ |
| **Infant outcomes** | |
| Development | Child development refers to how a child becomes able to do more complex things as they get older. Developmental milestones are a set of functional skills or age-specific tasks that most children can do at a certain age range.^7^ These skills include gross and fine motor, language, cognitive and social skills. |
| Lost to follow up | It was not possible to verify the status of the child at 12 months |
| **Favourable infant outcomes –** both of the following are required to state that an infant has a favourable outcome | |
| Thrive normally | If an infant gains weight following the normal trajectory according to the growth chart, the infant is said to be thriving normally. |
| Normal development | An infant is described as having normal development if they achieve the developmental milestones timeously. |
| **Unfavourable infant outcomes –** any of the following classify the child as having an unfavourable outcome | |
| Failure to thrive | The infant fails to maintain an established pattern of growth.^8^ |
| Delayed development | Developmental delay was defined as a significant delay in two or more of the following developmental domains: gross⁄fine motor, speech⁄language, social⁄personal, cognition, and activities of daily living.^9^ |
| Probable TB | An infant treated for TB following a laboratory diagnosis of TB or MDR/RR-TB or having the signs and symptoms of active TB disease. These include ‘any one of cough for more than 2 weeks, fever for more than 2 weeks or poor weight gain in past 3 months, or chest X-ray or both.^10^ |
| Neonatal death | Death of a live born infant in the first 28 days of life. An early neonatal death is a death which occurs in the first week of life.^11^ |
| Infant death | Infant dies before 12 months |

Abbreviations: MDR/RR-TB, multidrug/rifampicin-resistant TB; TB, tuberculosis; ART, antiretroviral therapy; Pre-XDR-TB, pre-extensively drug-resistant TB; XDR-TB, extensively drug-resistant TB; g, grams; WHO, World Health Organization

**Supplementary Table 2: The history of MDR/RR-TB treatment for pregnant women in South Africa**

| **Year** | **Regimen** |
| --- | --- |
| Prior to 2015 | All non-pregnant adults with MDR/RR-TB were treated with an 18 – 24-month regimen which included an injectable in accordance with the 2011 WHO guidelines and the 2013 South African DR-TB treatment guidelines:^12,13^   - 6 - 8 months injectable phase: KM, MFX, ETO, TRD, PZA - At least 18 months continuation phase: MFX, ETO, TRD, PZA^12^   In some cases, EMB and higher doses of INH were added.  **Pregnant women** may or may not have received MDR/RR-TB treatment, after “consideration of risks and benefits” by the clinician and the patient. No definitive regimen was recommended for pregnant women, and advice on the inclusion of injectable agents in a treatment regimen containing 3 or 4 oral drugs was contradictory. Ethionamide was advised to be given with caution, and the injectable agents were to be avoided “where possible”.  Bedaquiline was made available for global compassionate use in 2012, and access in South Africa was facilitated through the tightly regulated Bedaquiline Clinical Access Programme (BCAP) for selected patients at selected sites across the country between 2013 and 2015.^14^ Eligibility for bedaquiline was assessed on an individual case basis by the National Clinical Advisory Committee (NCAC) which either approved or rejected access to the drug. |
| 2015 | Introduction of a national policy framework to scale up the introduction of bedaquiline nationwide for all adults (≥ 18 years) diagnosed with pre-XDR or XDR-TB (i.e., MDR-TB that was resistant to fluoroquinolones and/or injectable agents), MDR-TB with both INH mutations (inhA and katG), and patients with MDR-TB and documented intolerance (i.e., hearing loss, renal dysfunction) to second-line TB treatment. ^15^  Bedaquiline could also be considered for other specific categories of patients with MDR-TB (e.g., < 18 years of age, **pregnant**, those in whom MDR-TB treatment had failed) following presentation by the treating clinician of individual patient cases to the Provincial or National Clinical Advisory Committees for approval.^15^  Most patients with MDR-TB, who did not meet the criteria above, continued to be offered the 18-24-month injectable-containing regimen. |
| 2017 | Introduction of the WHO-recommended shorter 9-11-month injectable-containing regimen for selected patients (those without previous exposure to second-line anti-TB drugs and those in whom resistance to FQs and injectables had been excluded).^16^  KM+CFZ+FQ+ETO+hINH+EMB+PZA  Notably, in South Africa, the national policy decision of substituting injectable agents with bedaquiline in selected patients (as described above) was also applied to eligible patients receiving the shorter 9-11-month regimen (including **pregnant women**).^17^  Patients not eligible for the shorter 9-11-month regimen continued to be offered longer regimens, with or without bedaquiline depending on eligibility. |
| 2018 | Introduction of a modified, 9-11-month, all-oral, bedaquiline-containing regimen as standard treatment for all patients with MDR-TB in South Africa.^18,19^  **All oral regimens:**^19^ *(for minimal disease)*  **9 – 11-month modified shorter regimen** which was no longer in line with the WHO guidelines.   - 4 – 6 months intensive phase: LZD+BDQ+hINH+LFX+CFZ+PZA+EMB - 5 months continuation phase: LFX+CFZ+PZA+EMB   **18-20-month longer regimens**: (*for patients not eligible for shorter regimens, i.e., those with previous exposure to any second-line anti-TB drugs, MDR/RR-TB with fluoroquinolone resistance, or complicated or extensive disease)*  These patients received a longer regimen tailored to their individual circumstances.^18^  **Pregnant women** with MDR/RR-TB were also eligible to receive the shorter, bedaquiline-containing 9–11-month regimen if they met the inclusion criteria, (i.e.: no prior history of treatment with second-line TB drugs (>1 month); MDR/RR-TB with no evidence of resistance to fluoroquinolones, BDQ, CFZ or LZD; only one INH mutation (*inhA* or *katG*) or no mutation causing INH resistance; no close contact with individuals with the above-mentioned characteristics; no evidence of complicated extra-pulmonary MDR/RR-TB [i.e., meningitis, pericarditis, osteoarticular, abdominal disease] or extensive, bilateral, cavitary pulmonary disease).^18^ |
| 2023 | On 1 September 2023, the South African National TB Department of Health implemented an adapted version of the WHO recommended 6-month BPaLM (BDQ+Pa+LZD+MFX) and BPaL (BDQ+Pa+LZD) regimens.^2^ The fluoroquinolone, moxifloxacin, was replaced with levofloxacin and the 6-month BPaLL (BDQ+Pa+LZD+LFX) regimen was recommended as standard treatment for non-pregnant people aged ≥ 15 years of age with MDR/RR-TB in South Africa.^2^  ***Pregnant women:*** Due to uncertainty regarding the reproductive toxicity of pretomanid, the BEAT Tuberculosis regimen approach was recommended for pregnant women with MDR/RR-TB.^20^ This approach allows pregnant women to receive an all oral 6-month regimen which includes delamanid instead of pretomanid. The BEAT Tuberculosis approach was to start a 5-drug regimen of BDQ+DLM+LZD+LFX+CFZ (BDLLC) and then drop either LFX or CFZ depending on the result of the fluoroquinolone DST so that:   - Pregnant women with MDR/RR-TB susceptible to the fluoroquinolones receive 6-months of: BDQ+DLM+LZD+LFX (BDLL) - Pregnant women with MDR/RR-TB resistant to the fluroquinolones receive 6-months of: BDQ+DLM+LZD (BDL)+CFZ - The BEAT Tuberculosis approach was followed in South Africa and included in the 2023 guidelines. In 2025 these regimens were included in WHO guidelines.^2,21^   **Pregnant women** with MDR/RR-TB who have extensive pulmonary disease (i.e., bilateral, cavitary with significant fibrosis, scarring or cavities in three or more lung zones) should have their treatment extended to nine months.  **Pregnant women** with MDR/RR-TB that is resistant to BDQ, Pa, DLM or LZD, or who have severe extra-pulmonary TB disease (i.e., meningitis, pericarditis, osteoarticular, abdominal or disseminated/miliary disease), or who are not responding to treatment, should be treated with a long-individualised regimen.^2^ |

Abbreviations: MDR/RR-TB, multidrug/rifampicin-resistant TB; TB, tuberculosis; WHO, World Health Organization; KM, kanamycin; MFX, moxifloxacin; ETO, ethionamide; TRD, terizidone; PZA , pyrazinamide; BDQ, bedaquiline; CFZ, clofazimine; FQ, fluoroquinolones; INH, isoniazid; hINH, high dose isoniazid; Pa, pretomanid; EMB, ethambutol; LZD, linezolid; LFX, levofloxacin; DLM, delamanid.

**REFERENCES**

1. World Health Organization. Meeting report of the WHO expert consultation on the definition of extensively drug-resistant tuberculosis, 27-29 October 2020. Geneva: 2021. CC BY-NC-SA 3.0 IGO.

2. South African National Department of Health. Clinical management of RR-TB: Updated Clinical Reference Guide. September 2023. Pretoria.

3. World Health Organization. Meeting report of the WHO expert consultation on drug-resistant tuberculosis treatment outcome definitions, 17-19 November 2020. Geneva: 2021. Licence: CC BY-NC-SA 3.0 IGO

4. World Health Organization, UNICEF. Low birthweight: country, regional and global estimates. Geneva: World Health Organization, 2004.

5. Van Niekerk E, Siebert I, Kruger T. An evidence-based approach to recurrent pregnancy loss . S Afr J OG 2013;19(3):61-65.

6. South African National Department of Health. Choice on Termination of Pregnancy Act. Pretoria 1996.

7. Berk LE. Young children: Prenatal through middle childhood. ISBN 0205011098. USA: Pearson, 2012.

8. The Free Medical Dictionary. <https://medical-dictionary.thefreedictionary.com/failure+to+thrive> (last accessed on 29 Nov 2017).

9. Riou EM, Ghosh S, Francoeur E, Shevell MI. Global developmental delay and its relationship to cognitive skills. Developmental medicine and child neurology 2009;51(8):600-6. (In eng). DOI: 10.1111/j.1469-8749.2008.03197.x.

10. World Health Organization. WHO operational handbook on tuberculosis. Module 5: Management of tuberculosis in children and adolescents. 2022. Licence: CC BY NC-SA 3.0 IGO. Geneva, Switzerland.

11. Stats South Africa. Perinatal deaths in South Africa 2014. Pretoria, South Africa: Statistics South Africa, 2016.

12. South African Department of Health. Management of drug-resistant tuberculosis. Policy Guidelines (updated - January 2013). Pretoria: Department of Health, 2013.

13. World Health Organization. Guidelines for the programmatic management of drug-resistant tuberculosis - 2011 Update. WHO/HTM/TB/2011.6. Geneva: 2011.

14. Conradie F, Meintjes G, Hughes J, et al. Clinical access to Bedaquiline Programme for the treatment of drug-resistant tuberculosis. S Afr Med J 2014;104(3):164-6. (In eng). DOI: 10.7196/samj.7263.

15. South African National Department of Health. Introduction of new drugs and drug regimens for the management of DR-TB in South Africa: Policy Framework. Pretoria: National Department of Health, 2015.

16. World Health Organization. WHO treatment guidelines for drug-resistant tuberculosis, 2016 update. WHO/HTM/TB/2016.04. Geneva 2016.

17. Ndjeka N, Hughes J, Reuter A, et al. Implementing novel regimens for drug-resistant TB in South Africa: what can the world learn? Int J Tuberc Lung Dis 2020;24(10):1073-1080. (In eng). DOI: 10.5588/ijtld.20.0174.

18. South African National Department of Health. Management of Rifampicin-Resistant Tuberculosis: A Clinical Reference Guide. November 2019. Last accessed 31 July 2025.

19. South African National Department of Health. Interim clinical guidance for the implementation of injectable-free regimens for rifampicin resistant tuberculosis in adults, adolescents and children. 2018. Pretoria, South Africa. <https://tbsouthafrica.org.za/resources/interim-clinical-guidance-implementation-injectable-free-regimens-rifampicin-resistant>.

20. Poswa A, Phillips, P, Badat,T, Khomeni, N, Jaylin, F, Rajaram, S, Koverjee, S, Conradie, F. . Enrollment of pregnant women with rifampicin-resistant tuberculosis (RR-TB) into BEAT Tuberculosis Randomized Clinical Trial: early outcomes (OA28-418-16). World Conference on Lung Health 2023. Paris 2023.

21. WHO consolidated guidelines on tuberculosis. Module 4: treatment and care. 2025. Licence: CC BY-NC-SA 3.0 IGO. Geneva: World Health Organization.
